# Long Non-Coding RNAs in Neuroblastoma: Pathogenesis, Biomarkers and Therapeutic Targets

**DOI:** 10.3390/ijms25115690

**Published:** 2024-05-23

**Authors:** Niels Vercouillie, Zhiyao Ren, Eva Terras, Tim Lammens

**Affiliations:** 1 Department of Internal Medicine and Pediatrics, Ghent University, 9000 Ghent, Belgium; niels.vercouillie@ugent.be (N.V.); zhiyao.ren@ugent.be (Z.R.); eva.terras@ugent.be (E.T.); 2 Department of Pediatric Hematology-Oncology and Stem Cell Transplantation, Ghent University Hospital, 9000 Ghent, Belgium; 3 Cancer Research Institute Ghent, 9000 Ghent, Belgium

**Keywords:** long non-coding RNA, neuroblastoma, cancer hallmark, diagnostic, prognostic, therapy

## Abstract

Neuroblastoma is the most common malignant extracranial solid tumor of childhood. Recent studies involving the application of advanced high-throughput “omics” techniques have revealed numerous genomic alterations, including aberrant coding-gene transcript levels and dysfunctional pathways, that drive the onset, growth, progression, and treatment resistance of neuroblastoma. Research conducted in the past decade has shown that long non-coding RNAs, once thought to be transcriptomic noise, play key roles in cancer development. With the recent and continuing increase in the amount of evidence for the underlying roles of long non-coding RNAs in neuroblastoma, the potential clinical implications of these RNAs cannot be ignored. In this review, we discuss their biological mechanisms of action in the context of the central driving mechanisms of neuroblastoma, focusing on potential contributions to the diagnosis, prognosis, and treatment of this disease. We also aim to provide a clear, integrated picture of future research opportunities.

## 1. Introduction

### 1.1. Neuroblastoma

Neuroblastoma, a malignant embryonic tumor of the sympathetic nervous system, is the most common extracranial childhood cancer worldwide [1]. Five-year overall survival (OS) rates for patients with neuroblastoma range widely from 60% to 98%, depending on risk group assignment. The rate for low-risk neuroblastoma requiring surgery alone is excellent (98%), whereas that for high-risk disease requiring intensive treatment consisting of chemotherapy, surgery, radiotherapy, stem cell transplantation, and immunotherapy is 60% at best [2,3,4,5]. Several clinical and biological factors have been shown to predict the clinical behavior of neuroblastoma. In this context, various stratification systems have been developed around the world to assign patients to low-, intermediate- and high-risk groups with corresponding treatment durations and intensities [3,6].

The diagnostic work-up for patients with suspected neuroblastoma should include blood and urine analyses. In 70–80% of neuroblastoma cases, urinary and serum catecholamine levels are elevated. Urinary vanillylmandelic and homovanillic acid levels are thus important parameters for the diagnosis of neuroblastoma [7,8]. This diagnosis is definitive with histological confirmation from biopsy samples. For localized tumors, bilateral lymph-node biopsy is recommended [9]. Given the prevalence of bone marrow involvement, bone marrow aspiration and trephine biopsy are also recommended [10]. Although primary tumor detection is important, the diagnosis of neuroblastoma can also be based on the histological positivity of a bone marrow biopsy or aspirate sample. Finally, various imaging methods are employed to obtain clear pictures of local and metastatic disease. Computed tomography (CT) and magnetic resonance imaging (MRI) are commonly performed to evaluate tumor spread. Whilst MRI is preferred for the examination of spinal involvement, CT is better for the detection of calcifications [9]. (^123^I-)metaiodobenzylguanidine scintigraphy is preferred for metastatic disease detection due to its high sensitivity and specificity, and can be supplemented with single-photon emission CT/CT for improved accuracy [2,11,12].

Neuroblastoma treatment is tailored according to the risk group. Surgical resection is indicated for patients with low-risk neuroblastoma, except for certain subgroups [13]. For intermediate-risk cases, treatment consists of neoadjuvant chemotherapy and surgery. The duration of neoadjuvant chemotherapy typically ranges from 6 to 24 weeks and is based on biological risk factors and the treatment response [14]. For cases in the highest risk group, treatment modalities include chemotherapy, surgery, radiotherapy, myeloablative therapy followed by stem cell transplantation, and immunotherapy. For detailed information on neuroblastoma treatment options, we refer the reader to the excellent review of Whittle et al. [13].

Several cytogenetic aberrations have been associated with poorer neuroblastoma prognoses. The amplification of *MYCN*, an oncogene located on chromosome 2p24–25, is one of the earliest discovered genetic markers of neuroblastoma and remains one of the strongest predictors of poor prognosis [15]. *MYCN* amplification correlates with faster tumor progression and advanced disease, which translates to poor event-free survival (EFS) and OS in children and infants with neuroblastoma [16]. It is associated with several segmental chromosomal aberrations, including chromosome 1p and 11q deletion and chromosome 17q amplification [17,18,19]. Similar to the *MYCN* status, DNA ploidy is a strong predictor of neuroblastoma prognosis [15]. Higher DNA indices generally correspond to lower staging, better treatment responses, and overall better prognoses, especially in the absence of *MYCN* amplification [20]. In contrast to chromosomal aberrations, very few gene mutations have been identified in neuroblastoma. *ALK*, *ATRX*, *PTPN11,* and *NRAS* mutations have been associated with the disease [21]. In addition, a group of patients has been recognized to have high *TERT* expression in the presence (or absence, in a small fraction) of *MYCN* or *TERT* rearrangements [22,23]. Finally, inactivating *ATRX* mutations accompanied by alternative lengthening of telomere pathway activation and low *TERT* mRNA levels have been identified in a high-risk subgroup lacking *MYCN* and *TERT* alterations [22,23].

In addition to changes at the DNA level, research has demonstrated significant gene expression changes at the mRNA level during neuroblastoma development. These alterations have revealed potential therapeutic targets, and expression signatures have been developed and shown to define prognosis [24,25]. Importantly, circulating mRNAs (as liquid biopsies) have been shown to predict poor survival in patients with high-risk neuroblastoma [26,27,28]. Despite the existing knowledge of the overall genetic and molecular properties of neuroblastoma, we have a very limited understanding of how these features contribute to the molecular events resulting in aggressive neuroblastoma cell biology. In addition, as 50% of high-risk neuroblastoma patients survive long-term on average, new parameters and biomarkers need to be identified for better risk stratification, and to gain further insights into the underlying biology of neuroblastoma.

### 1.2. Long Non-Coding RNAs

The majority of the human genome is transcribed, revealing the production of a wide spectrum of non-coding RNAs that are classified according to size: short RNAs are <200 nucleotides (nts) in length and include small interfering RNAs (siRNAs), piwi-interacting RNAs and microRNAs (miRNAs); long non-coding RNAs (lncRNAs) are longer than 200 nts and may comprise thousands of nucleotides [29]. Similar to other cancers, neuroblastoma has been reported to be characterized by changes in non-coding RNA, and especially miRNA, expression [30,31,32].

In recent years, lncRNAs have gained interest in the study of neuroblastoma pathogenesis. Although considered to be non-coding, some lncRNAs have translational potential, as small open reading frames can give rise to functional micropeptides [33]. LncRNAs can be classified according to their genome locations: (1) intronic lncRNAs are derived entirely from introns; (2) long intergenic non-coding RNAs (lincRNAs) are located between protein-coding regions; (3) antisense lncRNAs (or natural antisense transcripts) are located on the antisense strands of protein-coding genes, overlapping with exonic or intronic regions or covering the entire protein-coding sequence through an intron; (4) sense lncRNAs are located on the same strands as protein-coding genes, partially overlapping the genes or covering the entire sequence through an intron; (5) bidirectional lncRNAs transcribe in the opposite direction of promoters from protein-coding genes; and (6) enhancer RNAs are derived from transcriptional enhancers [34,35,36,37].

LncRNAs can also be grouped according to subcellular location. Classically, the division is made between nuclear lncRNAs, which are involved mainly in gene transcription and chromatin remodeling, and cytoplasmic lncRNAs, which are typically involved in the regulation of RNA-mediating functions and certain cell organelles. For an excellent review of lncRNA biology and function, we refer the reader to the recent publication of Statello and colleagues [34]. Briefly, nuclear lncRNAs may influence the transcriptional regulation of neighboring genes through the induction of R loops (DNA–lncRNA interactions) or triple helices (RNA–DNA–RNA interactions), or through the recruitment of chromatin looping and transcription factors [29,38,39]. It is becoming increasingly clear that lncRNAs facilitate these regulatory functions at least in part through their involvement in nuclear compartmentalization via phase separation. They concentrate and cluster at specific nuclear sites, interacting dynamically with diverse molecular partners to execute specialized functions [40]. In addition, nuclear lncRNAs have been found to be involved in alternative splicing. Nuclear and cytoplasmic lncRNAs have been shown to orchestrate post-transcriptional regulatory mechanisms including mRNA splicing, stability, and turnover due to their interactions with mRNA, miRNAs, and proteins. In this respect, lncRNAs have been described to act as sponges, sequestering specific proteins from target mRNAs or recruiting specific proteins to positively or negatively influence mRNA splicing and turnover. Additionally, cytoplasmic lncRNAs may interact with the machinery impacting protein translation, causing aberrant functioning that has been increasingly reported in diverse cancers [41]. Many cytoplasmic lncRNAs are also involved in the regulation of cell organelle function. Mitochondrial lncRNA, which can be transcriptionally nuclear or mitochondrial in origin (DNA), is often associated with mitochondrial metabolism, apoptosis, and crosstalk between mitochondria and nuclei [42]. Finally, exosomes contain lncRNA transcripts, and recent research has shown that exosome-associated lncRNAs are involved in cell epigenetic regulation, cell type reprogramming, and genomic instability [43].

**Figure 1:**
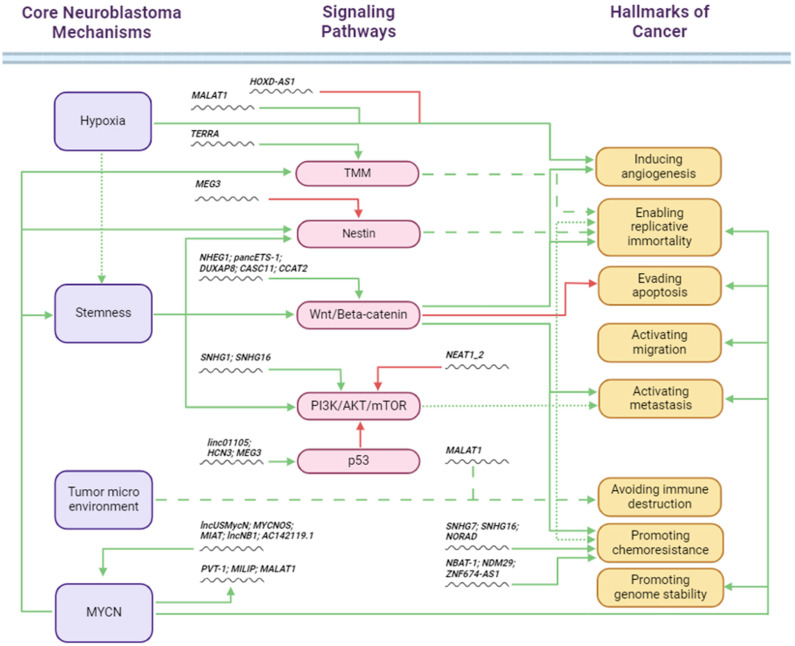
LncRNAs and hallmarks of neuroblastoma. This overview illustrates how central factors [*MYCN*, tumor hypoxia, tumor stemness, and TME cells (TAMs, TILs, and TMSCs)] mediate effects on neuroblastoma hallmarks through key signaling pathways (TMM = telomere maintenance mechanisms). The dysregulation of lncRNA expression and activity directly and indirectly impacts these hallmarks. Green arrows indicate promoting effects and red arrows indicate inhibitory effects. Created with BioRender.com.

Many lncRNAs have been shown to be aberrantly expressed in various cancers and to affect various biological processes, such as DNA damage, metastasis, immune evasion, treatment resistance, metabolic disturbances, and the maintenance of stem cell characteristics [44,45,46,47]. In this context, Modi and colleagues have recently shown, in an effort to assess the expression of lncRNAs in a range of pediatric leukemias and solid tumors, that neuroblastoma expresses an abundance of lncRNAs, many of which are specific for neuroblastoma disease [48]. Corroborating their computational methods, in vitro downregulation of TBX2-AS1, identified as a top candidate neuroblastoma lncRNA, inhibited neuroblastoma cell growth, illustrating the importance of lncRNA expression in neuroblastoma [48]. Here, we discuss the recent literature on the involvement of lncRNAs in the key processes of neuroblastoma development (Figure 1, Table 1), and suggest how this information can aid the design of novel diagnostic and prognostic tools and the development of novel therapeutics.

## 2. Long Non-Coding RNAs in Neuroblastoma

### 2.1. The MYCN Transcriptional Network

Several bidirectional interactions between *MYCN* and lncRNAs have been described; some lncRNAs regulate *MYCN* expression levels and others are regulated by *MYCN*. Most obviously, *MYCN* expression has been shown to be regulated by the lncRNA *MYCNOS*, which originates from the reverse-strand transcript encoding *MYCN* [49]. The lncRNA transcript *MYCNOS* directly modulates *MYCN* expression at the level of the *MYCN* promoter by functioning as a scaffold or by recruiting several proteins, including MAP4 and G3BP1, which form a complex that binds the *MYCN* promoter [49]. In addition to the full-length lncRNA, the MYCNOS peptide derived from it binds and inhibits glycogen synthase kinase 3β (GSK3β). As GSK3β promotes MYCN degradation, MYCNOS thus stabilizes *MYCN* expression levels. *MYCNOS* expression thus correlates positively with *MYCN* expression and, consequently, poor neuroblastoma outcomes [49]. *lncUSMycN* is an lncRNA that binds to the RNA-binding protein NonO, thereby increasing N-Myc expression through post-transcriptional processing. In silico data analysis has demonstrated that the upregulation of *lncUSMycN* is associated with enhanced cell proliferation and poorer OS in neuroblastoma [50,51].

Recent research has shown that increased expression levels of the lncRNA *myocardial infarction associated transcript* (*MIAT*) are associated with *MYCN* amplification in neuroblastoma tissue and cell lines [52]. *MIAT* and *MYCN* play a paired oncogene role in cell cycle control. *MIAT* acts as an upstream regulator of *MYC* oncogenes, but its expression is not regulated directly by these oncogenes. siRNA silencing of *MIAT* significantly induces apoptosis in cells with *MYCN* amplification, but only inhibits the growth of cells without such amplification. This reduced cell proliferation is due to the downregulation of downstream c-MYC genes, which causes cell cycle arrest in the G0/G1 and G2/M stages [52]. Similarly, *lncNB1* is highly overexpressed in *MYCN*-amplified neuroblastoma [53]. Binding between *lncNB1* and ribosomal protein L35 (RPL35) promotes the expression of E2F1, which in turn stimulates DEPDC1B gene expression. DEPDC1B, a GTPase-activating protein, then induces the phosphorylation of the ERK protein, leading to greater MYCN stability [53]. *LncNB1* knockdown inhibits the growth of neuroblastoma cells, leading to tumor regression. In sharp contrast, higher *lncNB1* and RPL35 expression levels have been associated with worse prognoses in patients with neuroblastoma [53].

The lncRNA *201 family member A* (*FAM201A*) was recently shown to encode a small peptide, NBASP, that suppresses the proliferation, migration, and invasion of neuroblastoma cells [54]. Immunoprecipitation identified fatty acid-binding protein 5 (FABP5) as an interaction partner of NBASP that had previously been already shown to be upregulated in MYCN-amplified neuroblastoma [54]. Through RNA sequencing and lipid metabolomics studies in cell lines overexpressing NBASP, followed by Western blot analyses (for ERK pathway), the authors could show that NBASP inhibits MAPK signaling [54]. This suggests that NBASP peptide delivery might be a therapeutic option to be explored.

A direct impact on transcriptional regulation of *MYCN* expression was reported by Rui and colleagues [56]. Analyzing the differential lncRNA expression between *MYCN*-amplified and *MYCN*-non-amplified cell lines and tissues, the lncRNA *AC142119.1* was identified to be strongly associated with *MYCN* amplification. *AC142119.1* was shown to recruit WDR5 to interact with the *MYCN* promotor, initiating the transcription of *MYCN* [56].

Recent research has demonstrated that the lncRNA *MILIP*, which is positively regulated by *MYCN*, is crucial in the orchestration of DNA double-strand break repair. Acting as an RNA scaffold, *MILIP* facilitates Ku70-Ku80 heterodimerization and thereby promotes non-homologous end joining [57]. Phenotypically, siRNA-mediated knockdown of *MILIP* resulted in a reduction in neuroblastoma cell viability through the induction of apoptosis and inhibition of cell proliferation. Importantly, it also made neuroblastoma cells more sensitive to DNA-damaging therapeutics.

The lncRNA *PVT-1* is also part of the *MYCN* transcriptional network in the context of neuroblastoma. MYCN proteins bind the promoter of *PVT-1* and thereby directly upregulates *PVT-1* gene expression [55]. *PVT-1* has been demonstrated to have an oncogenic role in several cancers, but its biological importance in neuroblastoma remains unclear [93].

The expression levels of several other lncRNAs [e.g., growth arrest-specific 5 (*GAS5*), *CCAT2,* and *SNHG1*] have been associated with the *MYCN* status, but direct evidence of their impacts on the *MYCN* transcriptional network remains lacking. The aberrant upregulation of *GAS5* is associated with reduced apoptosis and poorer cell survival [58]. Mazar et al. showed that the siRNA-mediated silencing of *GAS5* induces cell cycle arrest, reduced cell proliferation, and increased apoptosis in neuroblastoma cell lines in vitro [58]. *CCAT2*, an lncRNA upstream of the *MYCN* gene, is involved in the regulation of multiple molecular pathways, such as the phosphoinositide 3-kinase (PI3K)/protein kinase B (Akt), GSK3β/β-catenin, and Wnt/β-catenin pathways. Aberrant *CCAT2* expression has been associated with aberrant *MYCN* expression in neuroblastoma [61]. Similarly, increased *SNHG1* expression is associated with *MYCN* amplification. Increased expression of this lncRNA has been demonstrated in several cancers and associated with poor OS and EFS [59]. It has been implicated in cell proliferation and invasion and metastasis, with the underlying mechanism possibly relying on the sequestration of miR-338-3p by *SNHG1*, which consequently affects PLK4 levels and promotes epithelial–mesenchymal transition [60]. Sahu et al. proposed the use of the *SNHG1* expression level as a prognostic biomarker of clinical outcomes in patients with neuroblastoma [59]. In an effort to better characterize the lncRNAs involved in the spontaneous regression of neuroblastoma, Xinyao and colleagues identified a set of lncRNAs, differentiating between stage 4 patients who succumbed to the disease and stage 4S patients who survived [94]. Interestingly, several of the lncRNAs correlated to MYCN status, but no further mechanistic insights were provided [94]. Finally, through a large scale reanalysis of available RNA sequencing data, Modi and colleagues identified a group of adrenergic neuroblastoma lncRNAs, which are *MYCN*-associated, as the adrenergic state is known to be dependent on *MYCN* [48].

### 2.2. Neuroblastoma and Immune Evasion

Inflammation and immune evasion are fundamental for the progression of neuroblastoma [95]. Reciprocal communication between cancer cells and the tumor microenvironment (TME) contributes significantly to the immunosuppressive and pro-tumor nature of this microenvironment. Inflammatory and tumor-associated immune cells, aberrantly expressed immune checkpoint molecules, and locally aberrant extracellular matrix are involved in this communication. Remarkably, very little is known about the relationship between the TME and lncRNAs.

*MALAT1* is an lncRNA that contributes to the immune evasion of neuroblastoma cells. Mechanistically, increased *MALAT1* expression leads to ADAM10 expression via the *MALAT1*/*miR-92a*/ADAM10 axis, stimulating MICA/B secretion [80]. Increased MICA/B secretion is seen in the context of chemotherapy-induced senescence in neuroblastoma cells, and leads to increased MICA/B binding to surrounding natural killer cells, inhibiting these cells and allowing immune evasion [80]. Other functions of *MALAT1* are discussed below.

### 2.3. Neuroblastoma and Cancer Stem Cells

Neuroblastoma tumorigenesis is linked to the presence of cancer stem cells (CSCs). The origin and characteristics of these cells are not fully understood, although recent research has provided many new insights. For example, neuroblastoma-derived mesenchymal stem cells have been found to play a pro-tumoral role in the TME, promoting immune evasion and the invasion and metastasis of tumor cells [96]. The number of molecular elements identified as being linked mechanistically to stemness is also increasing [96,97,98]. Several signaling pathways, such as the PI3K/Akt/mammalian target of rapamycin (mTOR; PAM), Wnt, and RAS/RAF/MEK/ERK pathways, as well as dysregulated p53 signaling, are known to promote and maintain stemness in neuroblastoma. Recent research has also clarified that several lncRNAs are involved in these signaling pathways, promoting or inhibiting neuroblastoma tumorigenesis.

### 2.4. The PI3K/Akt/mTOR Signaling Pathway

The PI3K/AKT/mTOR (PAM) signaling pathway is crucial in health and disease, as it affects cellular functions such as proliferation, adhesion, migration, invasion, metabolism and survival [99]. LncRNAs have recently been shown to be involved in the regulation of the PAM pathway. *Small nucleolar RNA host gene 16* (*SNHG16*) is an lncRNA associated with poor neuroblastoma prognosis whose involvement in several other tumors and associated signaling pathways has been described [64]. In neuroblastoma, *SNHG16* mediates oncogenic effects via the PAM and RAS/RAF/MEK/ERK signaling pathways [63,100]. In the PAM signaling pathway, *SNHG16* sequesters *miR-338-3p*, which in turn is a key negative regulator of *PLK4* transcript levels [100]. The upregulation of PLK4, a key regulator of centriole duplication, activates the PAM signaling pathway, resulting in the stimulation of neuroblastoma cell proliferation, migration, and autophagy [65,100]. The lncRNA *SNHG1* also promotes tumorigenesis through a similar mechanism; it sequesters *miR-338-3p*, thereby increasing PLK4 levels [101]. The effects of increased PLK4 expression are again mediated through the PAM signaling pathway, contributing to a resistant neuroblastoma phenotype [65,101].

*Nuclear-enriched abundant transcript-I* (*NEAT1*) is an lncRNA composed of the lncRNA isoforms *NEAT1*_1 and *NEAT1*_2. The longer *NEAT1*_2 is responsible for paraspeckle functioning and inhibits PAM and ERK pathway activity. In doing so, it sequesters *miR-183-5p* of the *FOXP1* mRNA, upregulating FOXP1 and allowing it to mediate anti-oncogenic effects, leading to reduced proliferation and migration. *NEAT1*_2 downregulation in neuroblastoma has been demonstrated in vitro and in vivo, and is stronger in patients aged > 18 months and in advanced disease [66]. *NEAT1*_2 upregulation leads to the inhibition of neuroblastoma cell proliferation, migration, and invasion. In contrast, *NEAT1*_1 overexpression stimulates cell proliferation. Recently, Naveed et al. developed an antisense oligonucleotide (ASO) that, upon binding, causes the steric hindrance of the RNA polyadenylation of *NEAT1* in high-risk neuroblastoma [67]. This leads to “isoform switching”, where *NEAT1*_1 expression is downregulated and *NEAT1*_2 expression is upregulated [67].

### 2.5. p53 Signaling

p53 is known to inhibit the mTOR pathway under conditions of cellular stress in neuroblastoma through the transcription of proteins that negatively regulate the PAM pathway and thus induce cell cycle arrest, DNA repair, senescence, and apoptosis [60,102]. Increasing evidence indicates that lncRNAs can modulate p53 signaling, thereby indirectly affecting the PAM and RAS/RAF/MEK/ERK pathways. In this context, the lncRNAs *MEG3*, *HCN3,* and *linc01105* have been identified as relevant in neuroblastoma tumorigenesis: their expression levels are associated with International Neuroblastoma Staging System (INSS) staging [71]. Low and high expression levels of *linc01105* and *HCN3* are found in low- and high-stage neuroblastoma, respectively. *linc01105* and *HCN3* regulate apoptosis by interacting with Noxa and Bid, which are apoptosis-associated BCL-2 proteins that are part of the p53 pathway. The siRNA-mediated knockdown of *HCN3* and *linc01105* results in increased Noxa and decreased Bid expression, and thus increased apoptosis, in vitro. The simultaneous measurement of hypoxia-inducible factor-1α (HIF-1α) demonstrated that *linc01105* regulated proliferation via the translational modulation of this factor [71].

The lncRNA *MEG3* is considered to be an important tumor suppressor in neuroblastoma. *MEG3* overexpression is associated with the reduced expression of Bid and Noxa, as well as impaired cell growth and proliferation [71]. *MEG3* further mediates antitumor effects via the ubiquitin-mediated degradation of EZH2, and is negatively regulated by EZH2 via an undetermined mechanism. Nevertheless, *MEG3* is often downregulated in neuroblastoma, resulting in elevated EZH2 levels; in this context, it is associated with poor INSS staging and survival [72]. In contrast, in vitro *MEG3* overexpression induced the repression of proliferation, cell cycle arrest, and stem cell properties and the promotion of apoptosis in several neuroblastoma cell lines [70].

### 2.6. The Wnt/β-Catenin Pathway

The Wnt/β-catenin pathway is evolutionarily a highly conserved signaling cascade. The abnormal activation of Wnt/β-catenin signaling is, not surprisingly, associated strongly with the uncontrolled proliferation, metastasis, and angiogenesis of multiple cancers, including neuroblastoma [103]. Increased Wnt pathway activity is associated with chemoresistance, contributing significantly to the protective role of Wnt signaling in neuroblastoma stem cell populations [104,105]. In recent years, the number of lncRNAs demonstrated to affect the Wnt/β-catenin pathway has increased.

The upregulation of *NHEG1*, a nuclear lncRNA, has been shown to promote neuroblastoma cell aggressiveness, resulting in unfavorable OS and EFS. *NHEG1* binds and stabilizes DDX5, reducing this protein’s endogenous ubiquitination and proteasomal degradation and resulting in increased β-catenin activity [73]. The siRNA-mediated downregulation of *NHEG1* or DDX5 in murine xenograft models led to the differentiation and reduced growth and aggressiveness of neuroblastoma cells [73]. Recent research has shown that *NHEG1* additionally mediates oncogenic effects by acting as a ceRNA of *miR-665*, a regulator of HMGB1, a protein that promotes stemness in neuroblastoma [74,75]. *Ets-1 promoter-associated non-coding RNA* (*pancEts-1*) similarly promotes neuroblastoma progression by interacting with heterogeneous nuclear ribonucleoprotein K, which in turn interacts with β-catenin to inhibit its proteasome-mediated degradation, ultimately stabilizing β-catenin protein expression [76]. The lncRNA *double homeobox A pseudogene 8* (*DUXAP8*) also activates the Wnt/β-catenin pathway in neuroblastoma. Recent research has shown that it sequesters miR-29 from nucleolar protein 4 like (NOL4L), increasing NOL4L expression and stimulating tumor progression [77]. The stable knockdown of *DUXAP8* inhibits the growth of neuroblastoma and decreases the expression of proteins related to the Wnt pathway, including β-catenin, c-Myc, and cyclin D1 [77]. Although the impact of *DUXAP8*-mediated silencing on the Wnt/β-catenin pathway can be observed via NOL4L regulation, the mechanism by which NOL4L impacts the Wnt/β-catenin pathway remains unknown. The lncRNA *CASC11* acts similarly, sequestering *miR-676-3p* and reducing NOL4L expression [78]. Elevated *CASC11* expression levels have been found in neonatal neuroblastoma tissue and correlate positively with poor clinical outcomes. Conversely, the siRNA-mediated silencing of *CASC11* reduces the proliferation and invasiveness of neuroblastoma cells [78]. Finally, *CCAT2*, an lncRNA known to be involved in the genesis of many tumor types, exerts its physiological functions via the Wnt/β-catenin pathway [61]. In neuroblastoma, *CCAT2* upregulation is associated with decreased apoptosis and increased cell viability, proliferation, migration, and invasion [62,106].

### 2.7. Nestin

The lncRNA *MEG3* is generally considered to be an important suppressor of neuroblastoma tumorigenesis. It has various functions, including those related to the maintenance of CSC stemness. A recent analysis revealed that *MEG3* transcriptionally inhibits the expression of Nestin, a well-known CSC marker in neuroblastoma, potentially inhibiting CSC development [70]. The overexpression of Nestin is associated with aggressive neuroblastoma phenotypes [107].

### 2.8. Telomere Maintenance Mechanisms

Increased telomerase activity has been described in high-risk neuroblastoma, with a strong association between the presence of telomere maintenance mechanisms (TMMs) and tumor progression and mortality [108,109]. Furthermore, the presence of TMM correlates with more frequent relapse in patients with high-risk neuroblastoma, with the majority of relapse cases also exhibiting activation of TMMs [110]. Conversely, there is a clear association between low-risk neuroblastoma, which often undergoes spontaneous tumor regression, and the absence of TLM activation [111]. It is speculated that spontaneous regression in low-risk neuroblastoma takes place when telomere reserves are exhausted due to the lack of TMM and the inability to counteract telomere shortening pathways [111]. These findings underscore the potential prognostic and therapeutic significance of TMMs in neuroblastoma.

Several studies have revealed that *MYCN* amplification, ATRX inactivation, and *TERT* promoter rearrangement are crucial molecular mechanisms that drive TMMs in high-risk neuroblastoma [25,112,113,114,115,116]. In addition, evidence is also growing regarding the link between TMMs and lncRNAs. Recently, METTL3-driven RNA modification at the N6 position of internal adenosine (m6A) in *TERRA*, a telomere-derived lncRNA, was shown to be essential in telomere maintenance in ALT+ neuroblastoma cells [79]. Indeed, METTL3 knockdown cells had lower m6A modification in *TERRA*, compromising repair of telomeric DNA, as evidenced by an overall increase in γ-H2AX at the telomeres. Most importantly, treatment with bleomycin caused a continued increase in γ-H2AX in METTL3 knockdown cells. This indicates that METTL3 inhibitors might be a promising therapeutic approach in ALT+ neuroblastoma treatment [79].

### 2.9. Hypoxia and Angiogenesis

Hypoxia plays important roles in the development and maintenance of neuroblastoma stem cell characteristics, eliciting a series of adaptive mechanisms required for cell survival [96,97,117]. Mechanistically, it is becoming increasingly clear that these processes are mediated through the PAM signaling pathway, with the subsequent expression of HIF-1 and HIF-2, crucial to promote an immature neural-crest phenotype in neuroblastoma with self-renewable stem cell potential [96,97,117]. Hypoxia has also been linked to angiogenesis and vasculogenesis, regulated by known pro-angiogenic factors such as vascular endothelial cell growth factor (VEGF), platelet-derived growth factor, and fibroblast growth factor (FGF) [118]. A limited number of lncRNAs have been identified as being associated with hypoxia and/or angiogenesis.

The lncRNA *MALAT1* is associated with hypoxia and angiogenesis in the context of neuroblastoma, with expression levels increasing under hypoxic conditions [84]. *MALAT1* stimulates angiogenesis via the upregulation of FGF2 and VEGF [84]. VEGF has been identified as a factor that stimulates the survival, angiogenesis, and etoposide chemoresistance of neuroblastoma cells; recent studies conducted with murine xenograft models, however, have indicated that VEGF expression is not correlated with angiogenesis, but rather with the neural differentiation of neuroblastoma cells [119,120,121,122]. *MALAT1* also contributes to the maintenance of stemness, promoting immune evasion, migration, invasion, and metastasis [98]. These effects are mediated via the PAM pathway, the RAS/RAF/MEK/ERK pathway, and the tyrosine kinase receptor Axl. In addition, *MYCN* controls *MALAT1*; it regulates the histone demethylase JMJD1A, which in turn increases *MALAT1* expression at the transcriptional level [83]. As a result, *MYCN* overexpression leads to *MALAT1* overexpression, constituting another lncRNA-mediated mechanism by which *MYCN* contributes to neuroblastoma tumorigenesis [83]. In contrast, *MALAT1* silencing leads to the reduction of vascularization, migration, invasion, and neurite growth and is associated with the arrest of neuroblastoma cell differentiation due to RAS/RAF/MEK/ERK pathway inhibition [81,84]. *MALAT1* knockdown also leads to the aberrant activation of the peroxisome proliferator-activated receptor and the p53 signaling pathway [81]. *MALAT1* has been proposed as a diagnostic or prognostic biomarker for neuroblastoma and, given its important impact on tumorigenesis, potential clinical applications cannot be ignored [82,84].

The aberrant expression of the lncRNA *HOXD-AS1* has been demonstrated in various cancers, and *HOXD-AS1* downregulation has been associated with neuroblastoma tumorigenesis [123,124,125,126]. *HOXD-AS1* controls the expression of proteins involved in angiogenesis, inflammation, and hallmarks of metastatic cancer. Its expression may be regulated through the PAM pathway [85]. Yarmishyn et al. demonstrated that *HOXD-AS1* mediates the effects of retinoic acid treatment, leading to the differentiation and inhibited proliferation of neuroblastoma cells [85]. However, its expression is also increased in metastatic tumors, which seems contradictory to the upregulation of *HOXD-AS1* during cell differentiation [85]. Yarmishyn and colleagues have speculated that this is a compensatory response, with *HOXD-AS1* promoting the differentiation of metastatic neuroblastoma cell populations characterized by an overall loss of differentiation capacity [85]. Nevertheless, evidence for this hypothesis is currently lacking, and further research on the role of *HOXD-AS1* in neuroblastoma is needed.

### 2.10. Chemoresistance

Chemoresistance is a major obstacle in the treatment of high-risk neuroblastoma. In recent years, the number of lncRNAs identified to play key roles in the regulation of chemosensitivity has increased greatly. It is becoming increasingly clear that other molecular factors and signaling pathways are also involved in this process, as discussed above [96,97,98]. Many lncRNAs mediate their effects through these signaling pathways, indirectly influencing chemosensitivity. These factors contribute to the complexity of neuroblastoma chemoresistance.

The lncRNA *neuroblastoma-associated transcript 1* (*NBAT1*) is a tumor suppressor that regulates the subcellular localization of p53. Its downregulation has been described as an independent prognostic marker for high-risk neuroblastoma that leads to the cytoplasmic accumulation of p53, promoting chemoresistance [86]. *NBAT1* also mediates the effects of retinoic acid treatment. Finally, *NBAT1* regulates neural differentiation by inhibiting NRSF/REST expression, interacting with EZH2 to achieve the PRC2-dependent methylation of NRSF/REST promoters. The suppression of *NBAT1* is thus associated with the disruption of neural differentiation and increases in cell proliferation and invasion [87]. The difference in *NBAT1* expression between low- and high-risk neuroblastoma has been attributed to CpG methylation of its promoter and the presence of certain functional polymorphisms on chromosome 6p22 [127].

*NDM29* overexpression stimulates cisplatinum and doxorubicin cytotoxicity in neuroblastoma cell lines [88]. Mechanistically, *NDM29* suppresses the expression of multidrug resistance protein 1 (MRP1), an ATP-dependent transporter located in the plasma membrane that is involved in detoxification and chemoresistance [88]. The lncRNA *SNHG16* is also involved in neuroblastoma drug resistance. Its silencing leads to the reduced expression of MRP1 and multidrug resistance gene 1-type P-gp, as well as decreased cisplatinum resistance. *SNHG16* silencing also enhances cisplatinum cytotoxicity in vivo [100]. *SNHG7*, another lncRNA that contributes to neuroblastoma cisplatinum resistance, is associated with poor prognosis and overall survival [69]. Wang et al. showed that *SNHG7* regulates the *miR-329-3p*/MYO10 axis, thereby promoting chemoresistance through cisplatinum-induced autophagy [69]. *SNHG7* knockdown results in reduced cisplatinum resistance, migration, invasion, and glycolysis [68,69].

In addition, Kumming and colleagues highlighted the role of the lncRNA *ZNF674-AS1* in the regulation of cisplatin cytotoxicity. They showed that ZNF674-AS1 binds IGF2BP3, enhancing its binding capacity to Carbonic Anhydras 9 (CA9), which has been shown to enhance cisplatinum resistance [89].

The lncRNA *activated by DNA damage* (*NORAD*) is highly conserved and involved in genome stability and doxorubicin resistance. Research conducted with murine xenograft models showed that increased *NORAD* expression correlated negatively with the prognoses of patients with neuroblastoma [90]. *NORAD* upregulates the expression of histone deacetylase 8 (HDAC8) by sequestering its natural inhibitory miRNA *miR-144-3p*, thereby promoting neuroblastoma cell proliferation, metastasis, and doxorubicin resistance while inhibiting apoptosis and autophagy [90]. However, Yu and colleagues [92] reported the contradictory finding that low *NORAD* expression was associated with poor survival, *MYCN* amplification, and high-risk neuroblastoma. They showed that *NORAD* knockdown promoted cell proliferation and migration, leading to (G2/M-phase) cell cycle arrest and increased expression of PARP1, a DNA damage sensor [92]. Analysis of the underlying mechanisms revealed that *NORAD* silencing caused DNA damage due to the separation and reduced cohesion of sister chromatids [92]. In line with these results, Song et al. demonstrated that the overexpression of *NORAD* may lead to reduced 1-methyl-4-phenylpyridinium–induced cytotoxicity and apoptosis in SH-SY5Y neuroblastoma cells [91].

## 3. Discussion

In recent years, it has become increasingly clear that lncRNAs play critical roles in the genetic and epigenetic regulation of signaling pathways that define the hallmarks of neuroblastoma. Central mechanisms such as tumor hypoxia, tumor stemness, and TME (TAM, TIL, and TMSC) activities play significant roles in neuroblastoma tumorigenesis. Here, we present an integrated molecular network that provides more insight into the importance of lncRNAs and their connections to neuroblastoma hallmarks (Figure 1). An integrated approach is also crucial for the exploitation of the prognostic value and potential therapeutic opportunities provided by neuroblastoma lncRNAs.

The causes of the aberrant expression of certain lncRNAs in neuroblastoma remain unclear. We know, for example, that the hypermethylation of the promoter of *NBAT1* leads to low *NBAT1* expression in high-risk neuroblastoma [127], but the mechanisms underlying the disrupted expression of most other lncRNAs are not well understood. Segmental chromosomal aberrations may be involved in this process, but little is currently known and additional research is needed.

The lack of consistency in lncRNA nomenclature seriously hampers lncRNA research. Different authors refer to the same lncRNAs using different terms, making the integration of study data difficult. As the amount of data on lncRNAs in neuroblastoma continues to increase, this issue will become larger. Consensus nomenclature definitions are thus essential. lncipedia (https://lncipedia.org/) has taken a large step forward in this process.

Tumor biopsy remains the gold standard for the diagnosis of primary and metastatic neuroblastoma. However, neuroblastomas exhibit a high degree of intratumoral biological heterogeneity, and invasive tissue biopsies are often challenging to obtain. Significant research has been conducted in recent years on the diagnosis of neuroblastoma based on blood and bone marrow parameters [128]. The dissemination, necrosis, and secretion of tumor cells can be examined in blood and bone marrow [129,130]. Cell-free lncRNA is released by dying tumors and other cells [131], making lncRNAs potentially valuable biomarkers of tumor characteristics such as resistance to particular chemotherapeutic agents, encompassing tumor heterogeneity. In addition to freely circulating lncRNAs, lncRNAs can be detected in apoptotic bodies and exosomes, where they are shielded from extracellular RNases, making them more stable biomarkers [132]. LncRNAs could also be used to improve the detection of residual tumor tissue during treatment and follow-up.

Multidrug resistance is a major obstacle in the treatment of neuroblastoma, especially the high-risk form of the disease, and the significant roles of lncRNAs in this resistance are becoming increasingly clear. Several lncRNAs have been associated with resistance to doxorubicin, cisplatinum, adriamycin, and other chemotherapeutics [68,69,88,90,100,127]. For example, low *NBAT1* expression is associated with chemoresistance, suggesting that the *NBAT1* expression level could be used to predict treatment outcomes [86]. The relationships between many lncRNAs and neuroblastoma chemoresistance, however, remain unclear, emphasizing the need for further research. Also of interest would be the exploration of whether the determination of the expression levels of certain lncRNAs during the diagnostic evaluation of neuroblastoma could increase the accuracy of predictions for personalized therapeutic decision making. Finally, ncRNAs other than lncRNAs (mainly miRNAs and circular RNAs) may also play roles in resistance to multiple chemotherapeutic agents, but these roles are currently unclear [132].

The detection of aberrant lncRNA expression in neuroblastoma also provides opportunities for therapeutic innovation. As evidence that lncRNAs play significant roles in the pathogenesis of neuroblastoma accumulates, the use of ASO-based therapy for neuroblastoma appears to be a promising approach. The therapeutic efficacy of ASOs against various diseases, including amyotrophic lateral sclerosis, spinal muscular atrophy, and familial hypercholesterolemia, has been demonstrated [133,134,135,136]. Moreover, some neuroblastoma-associated lncRNAs are also crucial in the development of other neoplasms, adding to the value of new therapy development [137]. SiRNA-based approaches also appear to be promising, as siRNA-based lncRNA degradation has successfully reduced the expression of specific lncRNAs in some experimental studies [53,73,138,139]. However, as the majority of lncRNAs are located in cell nuclei, siRNA is often less effective than ASOs in targeting specific lncRNAs [140]. Although siRNA-based therapy is used for various conditions, the evidence for its effects on neuroblastoma is currently limited, and much of it derives from in vitro studies [141].

## 4. Conclusions

Research in the past decade has provided strong evidence that lncRNAs play important roles in neuroblastoma tumorigenesis and multidrug resistance. Nevertheless, evidence to support their use in clinical contexts remains insufficient. Thus, future research efforts are needed to (1) validate the prognostic value of lncRNAs in neuroblastoma, (2) disentangle their involvement in drug resistance pathways and determine their impacts on chemoresistance, and (3) exploit their therapeutic relevance.

## Figures and Tables

**Table 1:** Overview of neuroblastoma-associated lncRNAs.

lncRNA	Target/ Regulators	Function	Impact of Upregulation	Refs.
*MYCNOS*	*MYCN*	Increases *MYCN* expression through GSK3β inhibition and *MYCN* stabilization	Worse survival	[49]
*lncUSMycN*	*MYCNOS* NonO N-myc	Reduces *MYCN* expression by binding to NonO	Worse OS	[50,51]
*MIAT*	_	*MIAT* silencing reduces downstream c-MYC and *MYCN* expression, resulting in apoptosis (*MYCN*-A) and decreased cell proliferation (*MYCN*-NA)	-	[52]
*lncNB1*	E2F1 RPL35 DEPDC1B ERK n-Myc	Promotes E2F1 protein expression and stimulates N-Myc-driven oncogenesis; *lncNB1* knockdown leads to tumor regression	Worse OS	[53]
*FAM201A*	FABP5	*FAM201A* encodes NBASP which inhibits MAPK signaling.	-	[54]
*PVT-1*	_	Unknown in neuroblastoma	_	[55]
*AC142119.1*	*MYCN*	*AC142119.1* recruits WDR5 to interact with the *MYCN* promotor, initiating *MYCN* transcription	Worse survival	[56]
*MILIP*	Ku70 Ku80	Promotes non-homologous end joining by facilitating Ku70-Ku80 heterodimerization as an RNA scaffold; *MILIP* knockdown reduces neuroblastoma cell viability	_	[57]
*GAS5*	p53 BRCA1	*GAS5* silencing reduces cell proliferation and induces cell cycle arrest and apoptosis	Worse OS and EFS	[58]
*SNHG1*	*miR-338-3p*	Activates the PI3K/Akt pathway by sequestering *miR-338-3p* from PLK4; promotes neuroblastoma cell proliferation, migration and autophagy	Worse OS and EFS	[59,60]
*CCAT2*	p53	Inhibits proliferation and promotes apoptosis by regulating p53 and Bcl-2 proteins; may contribute to tumorigenesis via the Wnt/β-catenin pathway	Worse OS	[61,62]
*SNHG16*	*miR-338-3p*	Activates the PI3K/Akt pathway by sequestering *miR-338-3p* from PLK4; promotes neuroblastoma cell proliferation, migration and autophagy	Worse OS and EFS	[63,64,65]
*NEAT1*	*miR-183-5p*	*NEAT1*_2 sequesters *miR-183-5p*, leading to inhibition of the ERK/AKT pathway; its upregulation inhibits cell proliferation, migration and invasion *NEAT1*_1 stimulates proliferation	_	[66,67]
*SNHG7*	*miR-329-3p* MYO10	Stimulates chemoresistance through cisplatinum-induced autophagy; *SNHG7* knockdown reduces cisplatinum resistance, migration, invasion and glycolysis	Worse OS	[68,69]
*MEG3*	EZH2 (UCHL1)	Inhibits the expression of nestin, a CSC marker in neuroblastoma, and stimulates the ubiquitin-mediated degradation of EZH2; *MEG3* overexpression inhibits neuroblastoma growth in vivo and reduces Bid and Noxa expression	_	[70,71,72]
*NHEG1*	DDX6 *miR-665*	Reduces DDX5 degradation, thereby increasing β-catenin activity, and promotes cell aggressiveness by sequestering *miR-665* from HMGB1; *NHEG1* depletion leads to increased cell differentiation and reduced aggressiveness	Worse OS and EFS	[73,74,75]
*pancETS-1*	hnRNPK	Binds hnRNPK, allowing interaction with β-catenin and promoting proliferation, invasion and metastasis	Worse survival	[76]
*DUXAP8*	*miR-29*	Sequesters *miR-29* from NOL4L; *DUXAP8* knockdown inhibits growth and reduces downstream gene expression	Worse OS	[77]
*CASC11*	*miR-676-3p*	Sequesters *miR-676-3p* from NOL4L; *CASC11* silencing reduces neuroblastoma cell proliferation and invasiveness	Worse survival	[78]
*TERRA*	Telomerase	Interacts with telomerase, thereby enhancing telomere maintenance	-	[79]
*MALAT1*	AKT ERK1/2 Axl	*MALAT1* overexpression promotes tumor growth, invasion, migration and angiogenesis; *MALAT1* knockdown induces differentiation arrest, reduced neurite growth and abnormal PPAR and p53 activation.	_	[80,81,82,83,84]
*HOXD-AS1*	-	Controls the expression of proteins involved in angiogenesis, inflammation and hallmarks of metastatic cancer; mediates the effects of retinoic acid	_	[85]
*NBAT1*	-	Promotes neural differentiation via the NRSF/REST pathway and cytoplasmic p53 accumulation, leading to chemoresistance. *NBAT1* downregulation stimulates cell proliferation and invasion	Worse survival	[86,87]
*NDM29*	MDR1	Downregulates MDR1 expression, leading to chemoresistance; increased *NDM29* expression stimulates differentiation and inhibits cell cycle progression and proliferation	_	[88]
*ZNF674-AS1*	IGF2BF3	Binds IGF2BP3, thus enhancing its binding capacity to CA9 and consequently promoting cisplatinum resistance	Worse OS	[89]
*NORAD*	*miR-144-3p*	Sequesters *miR-144-3p* from HDAC; stimulates proliferation, tumor growth, metastasis and doxorubicin resistance; modulates the cohesion and separation of sister chromatids	_	[90,91,92]

OS = overall survival; EFS = event-free survival.

## Data Availability

Not applicable.

## References

[1] Maris J.M. (2010). Recent advances in neuroblastoma. N. Engl. J. Med..

[2] Newman E.A., Abdessalam S., Aldrink J.H., Austin M., Heaton T.E., Bruny J., Ehrlich P., Dasgupta R., Baertschiger R.M., Lautz T.B. (2019). Update on neuroblastoma. J. Pediatr. Surg..

[3] Irwin M.S., Naranjo A., Zhang F.F., Cohn S.L., London W.B., Gastier-Foster J.M., Ramirez N.C., Pfau R., Reshmi S., Wagner E. (2021). Revised neuroblastoma risk classification system: A report from the children’s oncology group. J. Clin. Oncol..

[4] Holmes K., Pötschger U., Pearson A.D., Sarnacki S., Cecchetto G., Gomez-Chacon J., Squire R., Freud E., Bysiek A., Matthyssens L.E. (2020). Influence of surgical excision on the survival of patients with stage 4 high-risk neuroblastoma: A report from the HR-NBL1/SIOPEN study. J. Clin. Oncol..

[5] Berthold F., Ernst A., Hero B., Klingebiel T., Kremens B., Schilling F.H., Simon T. (2018). Long-term outcomes of the GPOH NB97 trial for children with high-risk neuroblastoma comparing high-dose chemotherapy with autologous stem cell transplantation and oral chemotherapy as consolidation. Br. J. Cancer.

[6] Ambros P., Ambros I., Brodeur G., Haber M., Khan J., Nakagawara A., Schleiermacher G., Speleman F., Spitz R., London W. (2009). International consensus for neuroblastoma molecular diagnostics: Report from the International Neuroblastoma Risk Group (INRG) Biology Committee. Br. J. Cancer.

[7] Barco S., Gennai I., Reggiardo G., Galleni B., Barbagallo L., Maffia A., Viscardi E., De Leonardis F., Cecinati V., Sorrentino S. (2014). Urinary homovanillic and vanillylmandelic acid in the diagnosis of neuroblastoma: Report from the Italian Cooperative Group for Neuroblastoma. Clin. Biochem..

[8] Smith S.J., Diehl N.N., Smith B.D., Mohney B.G. (2010). Urine catecholamine levels as diagnostic markers for neuroblastoma in a defined population: Implications for ophthalmic practice. Eye.

[9] Swift C.C., Eklund M.J., Kraveka J.M., Alazraki A.L. (2018). Updates in diagnosis, management, and treatment of neuroblastoma. Radiographics.

[10] Rastogi P., Naseem S., Varma N., Das R., Ahluwalia J., Sachdeva M.U.S., Sharma P., Kumar N., Marwaha R.K. (2015). Bone marrow involvement in neuroblastoma: A study of hemato-morphological features. Indian J. Hematol. Blood Transfus..

[11] Brisse H.J., McCarville M.B., Granata C., Krug K.B., Wootton-Gorges S.L., Kanegawa K., Giammarile F., Schmidt M., Shulkin B.L., Matthay K.K. (2011). Guidelines for imaging and staging of neuroblastic tumors: Consensus report from the International Neuroblastoma Risk Group Project. Radiology.

[12] Sung A.J., Weiss B.D., Sharp S.E., Zhang B., Trout A.T. (2021). Prognostic significance of pretreatment 18F-FDG positron emission tomography/computed tomography in pediatric neuroblastoma. Pediatr. Radiol..

[13] Whittle S.B., Smith V., Doherty E., Zhao S., McCarty S., Zage P.E. (2017). Overview and recent advances in the treatment of neuroblastoma. Expert Rev. Anticancer Ther..

[14] Kaplan S.J., Holbrook C.T., McDaniel H.G., Buntain W.L., Crist W.M. (1980). Vasoactive intestinal peptide secreting tumors of childhood. Am. J. Dis. Child..

[15] Riley R.D., Heney D., Jones D.R., Sutton A.J., Lambert P.C., Abrams K.R., Young B., Wailoo A.J., Burchill S.A. (2004). A systematic review of molecular and biological tumor markers in neuroblastoma. Clin. Cancer Res..

[16] Otte J., Dyberg C., Pepich A., Johnsen J.I. (2021). MYCN function in neuroblastoma development. Front. Oncol..

[17] Attiyeh E.F., London W.B., Mossé Y.P., Wang Q., Winter C., Khazi D., McGrady P.W., Seeger R.C., Look A.T., Shimada H. (2005). Chromosome 1p and 11q deletions and outcome in neuroblastoma. N. Engl. J. Med..

[18] Bown N., Cotterill S., Łastowska M., O’Neill S., Pearson A.D., Plantaz D., Meddeb M., Danglot G., Brinkschmidt C., Christiansen H. (1999). Gain of chromosome arm 17q and adverse outcome in patients with neuroblastoma. N. Engl. J. Med..

[19] Caron H., van Sluis P., de Kraker J., Bökkerink J., Egeler M., Laureys G., Slater R., Westerveld A., Voute P., Versteeg R. (1996). Allelic loss of chromosome 1p as a predictor of unfavorable outcome in patients with neuroblastoma. N. Engl. J. Med..

[20] Park J.R., Eggert A., Caron H. (2010). Neuroblastoma: Biology, prognosis, and treatment. Hematol./Oncol. Clin. N. Am..

[21] Pugh T.J., Morozova O., Attiyeh E.F., Asgharzadeh S., Wei J.S., Auclair D., Carter S.L., Cibulskis K., Hanna M., Kiezun A. (2013). The genetic landscape of high-risk neuroblastoma. Nat. Genet..

[22] Peifer M., Hertwig F., Roels F., Dreidax D., Gartlgruber M., Menon R., Krämer A., Roncaioli J.L., Sand F., Heuckmann J.M. (2015). Telomerase activation by genomic rearrangements in high-risk neuroblastoma. Nature.

[23] Roderwieser A., Sand F., Walter E., Fischer J., Gecht J., Bartenhagen C., Ackermann S., Otte F., Welte A., Kahlert Y. (2019). Telomerase is a prognostic marker of poor outcome and a therapeutic target in neuroblastoma. JCO Precis. Oncol..

[24] Vermeulen J., De Preter K., Naranjo A., Vercruysse L., Van Roy N., Hellemans J., Swerts K., Bravo S., Scaruffi P., Tonini G.P. (2009). Predicting outcomes for children with neuroblastoma using a multigene-expression signature: A retrospective SIOPEN/COG/GPOH study. Lancet Oncol..

[25] Valentijn L.J., Koster J., Haneveld F., Aissa R.A., van Sluis P., Broekmans M.E., Molenaar J.J., van Nes J., Versteeg R. (2012). Functional MYCN signature predicts outcome of neuroblastoma irrespective of MYCN amplification. Proc. Natl. Acad. Sci. USA.

[26] van Zogchel L.M., Zappeij-Kannegieter L., Javadi A., Lugtigheid M., Gelineau N.U., Lak N.S., Zwijnenburg D.A., Koster J., Stutterheim J., van der Schoot C.E. (2021). Specific and sensitive detection of neuroblastoma mRNA markers by multiplex RT-qPCR. Cancers.

[27] Viprey V.F., Gregory W.M., Corrias M.V., Tchirkov A., Swerts K., Vicha A., Dallorso S., Brock P., Luksch R., Valteau-Couanet D. (2019). Neuroblastoma mRNAs predict outcome in children with stage 4 neuroblastoma: A European HR-NBL1/SIOPEN study. J. Clin. Oncol..

[28] Corrias M.V., Parodi S., Tchirkov A., Lammens T., Vicha A., Pasqualini C., Träger C., Yáñez Y., Dallorso S., Varesio L. (2018). Event-free survival of infants and toddlers enrolled in the HR-NBL-1/SIOPEN trial is associated with the level of neuroblastoma mRNAs at diagnosis. Pediatr. Blood Cancer.

[29] Fernandes J.C., Acuña S.M., Aoki J.I., Floeter-Winter L.M., Muxel S.M. (2019). Long non-coding RNAs in the regulation of gene expression: Physiology and disease. Non-Coding RNA.

[30] Morini M., Cangelosi D., Segalerba D., Marimpietri D., Raggi F., Castellano A., Fruci D., Font de Mora J., Cañete A., Yáñez Y. (2019). Exosomal microRNAs from longitudinal liquid biopsies for the prediction of response to induction chemotherapy in high-risk neuroblastoma patients: A proof of concept SIOPEN study‖. Cancers.

[31] De Preter K., Mestdagh P., Vermeulen J., Zeka F., Naranjo A., Bray I., Castel V., Chen C., Drozynska E., Eggert A. (2011). miRNA expression profiling enables risk stratification in archived and fresh neuroblastoma tumor samples. Clin. Cancer Res..

[32] Zeka F., Decock A., Van Goethem A., Vanderheyden K., Demuynck F., Lammens T., Helsmoortel H.H., Vermeulen J., Noguera R., Berbegall A.P. (2018). Circulating microRNA biomarkers for metastatic disease in neuroblastoma patients. JCI Insight.

[33] Hartford C.C.R., Lal A. (2020). When long noncoding becomes protein coding. Mol. Cell. Biol..

[34] Statello L., Guo C.-J., Chen L.-L., Huarte M. (2021). Gene regulation by long non-coding RNAs and its biological functions. Nat. Rev. Mol. Cell Biol..

[35] Derrien T., Johnson R., Bussotti G., Tanzer A., Djebali S., Tilgner H., Guernec G., Martin D., Merkel A., Knowles D.G. (2012). The GENCODE v7 catalog of human long noncoding RNAs: Analysis of their gene structure, evolution, and expression. Genome Res..

[36] Ma L., Bajic V.B., Zhang Z. (2013). On the classification of long non-coding RNAs. RNA Biol..

[37] Kopp F., Mendell J.T. (2018). Functional classification and experimental dissection of long noncoding RNAs. Cell.

[38] Martianov I., Ramadass A., Serra Barros A., Chow N., Akoulitchev A. (2007). Repression of the human dihydrofolate reductase gene by a non-coding interfering transcript. Nature.

[39] Mondal T., Subhash S., Vaid R., Enroth S., Uday S., Reinius B., Mitra S., Mohammed A., James A.R., Hoberg E. (2015). MEG3 long noncoding RNA regulates the TGF-β pathway genes through formation of RNA–DNA triplex structures. Nat. Commun..

[40] Somasundaram K., Gupta B., Jain N., Jana S. (2022). LncRNAs divide and rule: The master regulators of phase separation. Front. Genet..

[41] Hu G., Lou Z., Gupta M. (2014). The long non-coding RNA GAS5 cooperates with the eukaryotic translation initiation factor 4E to regulate c-Myc translation. PLoS ONE.

[42] Zhao Y., Liu S., Zhou L., Li X., Meng Y., Li Y., Li L., Jiao B., Bai L., Yu Y. (2019). Aberrant shuttling of long noncoding RNAs during the mitochondria-nuclear crosstalk in hepatocellular carcinoma cells. Am. J. Cancer Res..

[43] Fatima F., Nawaz M. (2017). Vesiculated long non-coding RNAs: Offshore packages deciphering trans-regulation between cells, cancer progression and resistance to therapies. Non-Coding RNA.

[44] Gutschner T., Diederichs S. (2012). The hallmarks of cancer: A long non-coding RNA point of view. RNA Biol..

[45] Fabris L., Juracek J., Calin G. (2020). Non-coding RNAs as cancer hallmarks in chronic lymphocytic leukemia. Int. J. Mol. Sci..

[46] Franco P.I.R., do Carmo Neto J.R., de Menezes L.B., Machado J.R., Miguel M.P. (2023). Revisiting the hallmarks of cancer: A new look at long noncoding RNAs in breast cancer. Pathol.-Res. Pract..

[47] Chi Y., Wang D., Wang J., Yu W., Yang J. (2019). Long non-coding RNA in the pathogenesis of cancers. Cells.

[48] Modi A., Lopez G., Conkrite K.L., Su C., Leung T.C., Ramanan S., Manduchi E., Johnson M.E., Cheung D., Gadd S. (2023). Integrative Genomic Analyses Identify LncRNA Regulatory Networks across Pediatric Leukemias and Solid Tumors. Cancer Res..

[49] Vadie N., Saayman S., Lenox A., Ackley A., Clemson M., Burdach J., Hart J., Vogt P.K., Morris K.V. (2015). MYCNOS functions as an antisense RNA regulating MYCN. RNA Biol..

[50] Liu P.Y., Erriquez D., Marshall G.M., Tee A.E., Polly P., Wong M., Liu B., Bell J.L., Zhang X.D., Milazzo G. (2014). Effects of a novel long noncoding RNA, lncUSMycN, on N-Myc expression and neuroblastoma progression. JNCI J. Natl. Cancer Inst..

[51] Liu P.Y., Atmadibrata B., Mondal S., Tee A.E., Liu T. (2016). NCYM is upregulated by lncUSMycN and modulates N-Myc expression. Int. J. Oncol..

[52] Feriancikova B., Feglarova T., Krskova L., Eckschlager T., Vicha A., Hrabeta J. (2021). MIAT Is an Upstream Regulator of NMYC and the Disruption of the MIAT/NMYC Axis Induces Cell Death in NMYC Amplified Neuroblastoma Cell Lines. Int. J. Mol. Sci..

[53] Liu P.Y., Tee A.E., Milazzo G., Hannan K.M., Maag J., Mondal S., Atmadibrata B., Bartonicek N., Peng H., Ho N. (2019). The long noncoding RNA lncNB1 promotes tumorigenesis by interacting with ribosomal protein RPL35. Nat. Commun..

[54] Ye M., Gao R., Chen S., Bai J., Chen J., Lu F., Gu D., Shi X., Yu P., Tian Y. (2023). FAM201A encodes small protein NBASP to inhibit neuroblastoma progression via inactivating MAPK pathway mediated by FABP5. Commun. Biol..

[55] Carramusa L., Contino F., Ferro A., Minafra L., Perconti G., Giallongo A., Feo S. (2007). The PVT-1 oncogene is a Myc protein target that is overexpressed in transformed cells. J. Cell. Physiol..

[56] Yang R., Liu N., Li T., Liu F., Zhang J., Zhao H., Zou L., He X. (2023). LncRNA AC142119. 1 facilitates the progression of neuroblastoma by epigenetically initiating the transcription of MYCN. J. Transl. Med..

[57] Wang P.L., Teng L., Feng Y.C., Yue Y.M., Han M.M., Yan Q., Ye K., Tang C.X., Zhang S.N., Fei Qi T. (2022). The N-Myc-responsive lncRNA MILIP promotes DNA double-strand break repair through non-homologous end joining. Proc. Natl. Acad. Sci. USA.

[58] Mazar J., Rosado A., Shelley J., Marchica J., Westmoreland T.J. (2017). The long non-coding RNA GAS5 differentially regulates cell cycle arrest and apoptosis through activation of BRCA1 and p53 in human neuroblastoma. Oncotarget.

[59] Sahu D., Hsu C.-L., Lin C.-C., Yang T.-W., Hsu W.-M., Ho S.-Y., Juan H.-F., Huang H.-C. (2016). Co-expression analysis identifies long noncoding RNA SNHG1 as a novel predictor for event-free survival in neuroblastoma. Oncotarget.

[60] Tian X., Zhou D., Chen L., Tian Y., Zhong B., Cao Y., Dong Q., Zhou M., Yan J., Wang Y. (2018). Polo-like kinase 4 mediates epithelial–mesenchymal transition in neuroblastoma via PI3K/Akt signaling pathway. Cell Death Dis..

[61] Ma S., Wang W., Zhang D., Zhao G., Lu Z. (2022). Long non-coding RNA colon cancer-associated transcript 2: Role and function in human cancers. Chin. Med. J..

[62] Chen M., Zhao M., Hou Y., Zhu B. (2021). Expression of lncRNA CCAT2 in children with neuroblastoma and its effect on cancer cell growth. Mol. Cell. Biochem..

[63] Deng D., Yang S., Wang X. (2020). Long non-coding RNA SNHG16 regulates cell behaviors through miR-542-3p/HNF4α axis via RAS/RAF/MEK/ERK signaling pathway in pediatric neuroblastoma cells. Biosci. Rep..

[64] Gong C.-Y., Tang R., Nan W., Zhou K.-S., Zhang H.-H. (2020). Role of SNHG16 in human cancer. Clin. Chim. Acta.

[65] Wen Y., Gong X., Dong Y., Tang C. (2020). Long non coding RNA SNHG16 facilitates proliferation, migration, invasion and autophagy of neuroblastoma cells via sponging miR-542-3p and upregulating ATG5 expression. OncoTargets Ther..

[66] Pan W., Wu A., Yu H., Yu Q., Zheng B., Yang W., Tian D., Gao Y., Li P. (2020). NEAT1 Negatively regulates cell proliferation and migration of neuroblastoma cells by miR-183-5p/FOXP1 Via the ERK/AKT pathway. Cell Transplant..

[67] Naveed A., Cooper J.A., Li R., Hubbard A., Chen J., Liu T., Wilton S.D., Fletcher S., Fox A.H. (2021). NEAT1 polyA-modulating antisense oligonucleotides reveal opposing functions for both long non-coding RNA isoforms in neuroblastoma. Cell. Mol. Life Sci..

[68] Jia J., Zhang D., Zhang J., Yang L., Zhao G., Yang H., Wang J. (2020). Long non-coding RNA SNHG7 promotes neuroblastoma progression through sponging miR-323a-5p and miR-342-5p. Biomed. Pharmacother..

[69] Wang S., Wang X., Zhang C. (2020). LncRNA SNHG7 enhances chemoresistance in neuroblastoma through cisplatin-induced autophagy by regulating miR-329-3p/MYO10 axis. Eur. Rev. Med. Pharmacol. Sci..

[70] Ye M., Wei M., Gao R., Dong K. (2022). Upregulation of MEG3 inhibits neuroblastoma progression via decreasing proliferation and promoting apoptosis. Life Res. J..

[71] Tang W., Dong K., Li K., Dong R., Zheng S. (2016). MEG3, HCN3 and linc01105 influence the proliferation and apoptosis of neuroblastoma cells via the HIF-1α and p53 pathways. Sci. Rep..

[72] Ye M., Gao R., Chen S., Wei M., Wang J., Zhang B., Wu S., Xu Y., Wu P., Chen X. (2022). Downregulation of MEG3 and upregulation of EZH2 cooperatively promote neuroblastoma progression. J. Cell. Mol. Med..

[73] Zhao X., Li D., Yang F., Lian H., Wang J., Wang X., Fang E., Song H., Hu A., Guo Y. (2020). Long noncoding RNA NHEG1 Drives β-catenin transactivation and neuroblastoma progression through interacting with DDX5. Mol. Ther..

[74] Yan D., Ma Z., Liu C., Wang C., Deng Y., Liu W., Xu B. (2019). Corynoxine B ameliorates HMGB1-dependent autophagy dysfunction during manganese exposure in SH-SY5Y human neuroblastoma cells. Food Chem. Toxicol..

[75] Zhang Y., Hu Y., Pan A., He L., Wang J., Zhou F., Lei Y., Wang Y. (2021). Long non-coding RNA NHEG1/hsa-miR-665/HMGB1 axis is involved in the regulation of neuroblastoma progression. Bioengineered.

[76] Li D., Wang X., Mei H., Fang E., Ye L., Song H., Yang F., Li H., Huang K., Zheng L. (2018). Long noncoding RNA pancEts-1 promotes neuroblastoma progression through hnRNPK-mediated β-catenin stabilization. Cancer Res..

[77] Nie L., Li C., Zhao T., Wang Y., Liu J. (2020). LncRNA double homeobox A pseudogene 8 (DUXAP8) facilitates the progression of neuroblastoma and activates Wnt/β-catenin pathway via microRNA-29/nucleolar protein 4 like (NOL4L) axis. Brain Res..

[78] Yu Z., Zhang J., Han J. (2020). Silencing CASC11 curbs neonatal neuroblastoma progression through modulating microRNA-676-3p/nucleolar protein 4 like (NOL4L) axis. Pediatr. Res..

[79] Vaid R., Thombare K., Mendez A., Burgos-Panadero R., Djos A., Jachimowicz D., Lundberg K.I., Bartenhagen C., Kumar N., Tümmler C. (2024). METTL3 drives telomere targeting of TERRA lncRNA through m6A-dependent R-loop formation: A therapeutic target for ALT-positive neuroblastoma. Nucleic Acids Res..

[80] Zhang Y., Hu R., Xi B., Nie D., Xu H., Liu A. (2022). Mechanisms of Senescence-Related NKG2D Ligands Release and Immune Escape Induced by Chemotherapy in Neuroblastoma Cells. Front. Cell Dev. Biol..

[81] Chen L., Feng P., Zhu X., He S., Duan J., Zhou D. (2016). Long non-coding RNA Malat1 promotes neurite outgrowth through activation of ERK/MAPK signalling pathway in N2a cells. J. Cell. Mol. Med..

[82] Li Z.-X., Zhu Q.-N., Zhang H.-B., Hu Y., Wang G., Zhu Y.-S. (2018). MALAT1: A potential biomarker in cancer. Cancer Manag. Res..

[83] Tee A.E., Ling D., Nelson C., Atmadibrata B., Dinger M.E., Xu N., Mizukami T., Liu P.Y., Liu B., Cheung B. (2014). The histone demethylase JMJD1A induces cell migration and invasion by up-regulating the expression of the long noncoding RNA MALAT1. Oncotarget.

[84] Tee A.E., Liu B., Song R., Li J., Pasquier E., Cheung B.B., Jiang C., Marshall G.M., Haber M., Norris M.D. (2016). The long noncoding RNA MALAT1 promotes tumor-driven angiogenesis by up-regulating pro-angiogenic gene expression. Oncotarget.

[85] Yarmishyn A.A., Batagov A.O., Tan J.Z., Sundaram G.M., Sampath P., Kuznetsov V.A., Kurochkin I.V. (2014). HOXD-AS1 is a novel lncRNA encoded in HOXD cluster and a marker of neuroblastoma progression revealed via integrative analysis of noncoding transcriptome. BMC Genom..

[86] Mitra S., Muralidharan S.V., Di Marco M., Juvvuna P.K., Kosalai S.T., Reischl S., Jachimowicz D., Subhash S., Raimondi I., Kurian L. (2021). Subcellular Distribution of p53 by the p53-Responsive lncRNA NBAT1 Determines Chemotherapeutic Response in NeuroblastomaThe p53 Responsive lncRNA NBAT1 Determines Chemoresistance. Cancer Res..

[87] Pandey G.K., Mitra S., Subhash S., Hertwig F., Kanduri M., Mishra K., Fransson S., Ganeshram A., Mondal T., Bandaru S. (2014). The risk-associated long noncoding RNA NBAT-1 controls neuroblastoma progression by regulating cell proliferation and neuronal differentiation. Cancer Cell.

[88] Garbati P., Barbieri R., Cangelosi D., Zanon C., Costa D., Eva A., Thellung S., Calderoni M., Baldini F., Tonini G.P. (2020). MCM2 and carbonic anhydrase 9 are novel potential targets for neuroblastoma pharmacological treatment. Biomedicines.

[89] Zhao K., Wang X., Jin Y., Zhu X., Zhou T., Yu Y., Ji X., Chang Y., Luo J., Ni X. (2024). LncRNA ZNF674-AS1 drives cell growth and inhibits cisplatin-induced pyroptosis via up-regulating CA9 in neuroblastoma. Cell Death Dis..

[90] Wang B., Xu L., Zhang J., Cheng X., Xu Q., Wang J., Mao F. (2020). LncRNA NORAD accelerates the progression and doxorubicin resistance of neuroblastoma through up-regulating HDAC8 via sponging miR-144-3p. Biomed. Pharmacother..

[91] Song Q., Geng Y., Li Y., Wang L., Qin J. (2019). Long noncoding RNA NORAD regulates MPP+-induced Parkinson’s disease model cells. J. Chem. Neuroanat..

[92] Yu Y., Chen F., Jin Y., Yang Y., Wang S., Zhang J., Chen C., Zeng Q., Han W., Wang H. (2020). Downregulated NORAD in neuroblastoma promotes cell proliferation via chromosomal instability and predicts poor prognosis. Acta Biochim. Pol..

[93] Onagoruwa O.T., Pal G., Ochu C., Ogunwobi O.O. (2020). Oncogenic role of PVT1 and therapeutic implications. Front. Oncol..

[94] Meng X., Fang E., Zhao X., Feng J. (2020). Identification of prognostic long noncoding RNAs associated with spontaneous regression of neuroblastoma. Cancer Med..

[95] Nallasamy P., Chava S., Verma S.S., Mishra S., Gorantla S., Coulter D.W., Byrareddy S.N., Batra S.K., Gupta S.C., Challagundla K.B. (2018). PD-L1, inflammation, non-coding RNAs, and neuroblastoma: Immuno-oncology perspective. Seminars in Cancer Biology.

[96] Veschi V., Verona F., Thiele C.J. (2019). Cancer stem cells and neuroblastoma: Characteristics and therapeutic targeting options. Front. Endocrinol..

[97] Bahmad H.F., Chamaa F., Assi S., Chalhoub R.M., Abou-Antoun T., Abou-Kheir W. (2019). Cancer stem cells in neuroblastoma: Expanding the Therapeutic Frontier. Front. Mol. Neurosci..

[98] Farina A.R., Cappabianca L.A., Zelli V., Sebastiano M., Mackay A.R. (2021). Mechanisms involved in selecting and maintaining neuroblastoma cancer stem cell populations, and perspectives for therapeutic targeting. World J. Stem Cells.

[99] Bader A.G., Kang S., Zhao L., Vogt P.K. (2005). Oncogenic PI3K deregulates transcription and translation. Nat. Rev. Cancer.

[100] Xu Z., Sun Y., Wang D., Sun H., Liu X. (2020). SNHG16 promotes tumorigenesis and cisplatin resistance by regulating miR-338-3p/PLK4 pathway in neuroblastoma cells. Cancer Cell Int..

[101] Zhang N., Liu F., Ma T., Zeng Z., Zhang J. (2019). LncRNA SNHG1 contributes to tumorigenesis and mechanism by targeting miR-338-3p to regulate PLK4 in human neuroblastoma. Eur. Rev. Med. Pharmacol. Sci..

[102] Moreno-Smith M., Lakoma A., Chen Z., Tao L., Scorsone K.A., Schild L., Aviles-Padilla K., Nikzad R., Zhang Y., Chakraborty R. (2017). p53 Nongenotoxic Activation and mTORC1 Inhibition Lead to Effective Combination for Neuroblastoma Therapyp53 Activation and mTORC1 Inhibition in Neuroblastoma. Clin. Cancer Res..

[103] He L., Zhou H., Zeng Z., Yao H., Jiang W., Qu H. (2019). Wnt/β-catenin signaling cascade: A promising target for glioma therapy. J. Cell. Physiol..

[104] Kunnimalaiyaan S., Schwartz V.K., Jackson I.A., Clark Gamblin T., Kunnimalaiyaan M. (2018). Antiproliferative and apoptotic effect of LY2090314, a GSK-3 inhibitor, in neuroblastoma in vitro. BMC Cancer.

[105] Vangipuram S.D., Buck S.A., Lyman W.D. (2012). Wnt pathway activity confers chemoresistance to cancer stem-like cells in a neuroblastoma cell line. Tumor Biol..

[106] Fan Y.-H., Fang H., Ji C.-X., Xie H., Xiao B., Zhu X.-G. (2017). Long noncoding RNA CCAT2 can predict metastasis and poor prognosis: A meta-analysis. Clin. Chim. Acta.

[107] Thomas S.K., Messam C.A., Spengler B.A., Biedler J.L., Ross R.A. (2004). Nestin is a potential mediator of malignancy in human neuroblastoma cells. J. Biol. Chem..

[108] Ohali A., Avigad S., Ash S., Goshen Y., Luria D., Feinmesser M., Zaizov R., Yaniv I. (2006). Telomere length is a prognostic factor in neuroblastoma. Cancer.

[109] Pezzolo A., Pistorio A., Gambini C., Haupt R., Ferraro M., Erminio G., De Bernardi B., Garaventa A., Pistoia V. (2015). Intratumoral diversity of telomere length in individual neuroblastoma tumors. Oncotarget.

[110] Koneru B., Lopez G., Farooqi A., Conkrite K.L., Nguyen T.H., Macha S.J., Modi A., Rokita J.L., Urias E., Hindle A. (2020). Telomere maintenance mechanisms define clinical outcome in high-risk neuroblastoma. Cancer Res..

[111] Yu E.Y., Cheung N.-K.V., Lue N.F. (2022). Connecting telomere maintenance and regulation to the developmental origin and differentiation states of neuroblastoma tumor cells. J. Hematol. Oncol..

[112] Kuzyk A., Gartner J., Mai S. (2016). Identification of neuroblastoma subgroups based on three-dimensional telomere organization. Transl. Oncol..

[113] Dagg R.A., Pickett H.A., Neumann A.A., Napier C.E., Henson J.D., Teber E.T., Arthur J.W., Reynolds C.P., Murray J., Haber M. (2017). Extensive proliferation of human cancer cells with ever-shorter telomeres. Cell Rep..

[114] Zeineldin M., Federico S., Chen X., Fan Y., Xu B., Stewart E., Zhou X., Jeon J., Griffiths L., Nguyen R. (2020). MYCN amplification and ATRX mutations are incompatible in neuroblastoma. Nat. Commun..

[115] Hu Y., Shi G., Zhang L., Li F., Jiang Y., Jiang S., Ma W., Zhao Y., Songyang Z., Huang J. (2016). Switch telomerase to ALT mechanism by inducing telomeric DNA damages and dysfunction of ATRX and DAXX. Sci. Rep..

[116] Westermann F., Muth D., Benner A., Bauer T., Henrich K.-O., Oberthuer A., Brors B., Beissbarth T., Vandesompele J., Pattyn F. (2008). Distinct transcriptional MYCN/c-MYC activities are associated with spontaneous regression or malignant progression in neuroblastomas. Genome Biol..

[117] Påhlman S., Mohlin S. (2018). Hypoxia and hypoxia-inducible factors in neuroblastoma. Cell Tissue Res..

[118] Borriello L., Seeger R.C., Asgharzadeh S., DeClerck Y.A. (2016). More than the genes, the tumor microenvironment in neuroblastoma. Cancer Lett..

[119] Beierle E.A., Nagaram A., Dai W., Iyengar M., Chen M.K. (2005). VEGF-mediated survivin expression in neuroblastoma cells. J. Surg. Res..

[120] Zhang Z.-C., Tang C., Dong Y., Zhang J., Yuan T., Tao S.-C., Li X.-L. (2017). Targeting the long noncoding RNA MALAT1 blocks the pro-angiogenic effects of osteosarcoma and suppresses tumour growth. Int. J. Biol. Sci..

[121] Das B., Yeger H., Tsuchida R., Torkin R., Gee M.F., Thorner P.S., Shibuya M., Malkin D., Baruchel S. (2005). A hypoxia-driven vascular endothelial growth factor/Flt1 autocrine loop interacts with hypoxia-inducible factor-1α through mitogen-activated protein kinase/extracellular signal-regulated kinase 1/2 pathway in neuroblastoma. Cancer Res..

[122] Weng W.-C., Lin K.-H., Wu P.-Y., Ho Y.-H., Liu Y.-L., Wang B.-J., Chen C.-C., Lin Y.-C., Liao Y.-F., Lee W.-T. (2017). VEGF expression correlates with neuronal differentiation and predicts a favorable prognosis in patients with neuroblastoma. Sci. Rep..

[123] Takeuchi T., Tomida S., Yatabe Y., Kosaka T., Osada H., Yanagisawa K., Mitsudomi T., Takahashi T. (2006). Expression profile–defined classification of lung adenocarcinoma shows close relationship with underlying major genetic changes and clinicopathologic behaviors. J. Clin. Oncol..

[124] Champine P.J., Michaelson J., Weimer B.C., Welch D.R., DeWald D.B. (2007). Microarray analysis reveals potential mechanisms of BRMS1-mediated metastasis suppression. Clin. Exp. Metastasis.

[125] Lee D.C., Kang Y.K., Kim W.H., Jang Y.J., Kim D.J., Park I.Y., Sohn B.H., Sohn H.A., Lee H.G., Lim J.S. (2008). Functional and clinical evidence for NDRG2 as a candidate suppressor of liver cancer metastasis. Cancer Res..

[126] Sabates-Bellver J., Van der Flier L.G., de Palo M., Cattaneo E., Maake C., Rehrauer H., Laczko E., Kurowski M.A., Bujnicki J.M., Menigatti M. (2007). Transcriptome profile of human colorectal adenomas. Mol. Cancer Res..

[127] Pandey G.K., Kanduri C. (2015). Long noncoding RNAs and neuroblastoma. Oncotarget.

[128] Merugu S., Chen L., Gavens E., Gabra H., Brougham M., Makin G., Ng A., Murphy D., Gabriel A.S., Robinson M.L. (2020). Detection of circulating and disseminated neuroblastoma cells using the ImageStream flow cytometer for use as predictive and pharmacodynamic biomarkers. Clin. Cancer Res..

[129] Lodrini M., Graef J., Thole-Kliesch T.M., Astrahantseff K., Sprüssel A., Grimaldi M., Peitz C., Linke R.B., Hollander J.F., Lankes E. (2022). Targeted analysis of cell-free circulating tumor DNA is suitable for early relapse and actionable target detection in patients with neuroblastoma. Clin. Cancer Res..

[130] Ye Q., Ling S., Zheng S., Xu X. (2019). Liquid biopsy in hepatocellular carcinoma: Circulating tumor cells and circulating tumor DNA. Mol. Cancer.

[131] Weiser D.A., West-Szymanski D.C., Fraint E., Weiner S., Rivas M.A., Zhao C.W., He C., Applebaum M.A. (2019). Progress toward liquid biopsies in pediatric solid tumors. Cancer Metastasis Rev..

[132] Huang S., Gong N., Li J., Hong M., Li L., Zhang L., Zhang H. (2022). The role of ncRNAs in neuroblastoma: Mechanisms, biomarkers and therapeutic targets. Biomark. Res..

[133] Miller T., Cudkowicz M., Shaw P.J., Andersen P.M., Atassi N., Bucelli R.C., Genge A., Glass J., Ladha S., Ludolph A.L. (2020). Phase 1–2 trial of antisense oligonucleotide tofersen for SOD1 ALS. N. Engl. J. Med..

[134] Benatar M., Wuu J., Andersen P.M., Bucelli R.C., Andrews J.A., Otto M., Farahany N.A., Harrington E.A., Chen W., Mitchell A.A. (2022). Design of a randomized, placebo-controlled, phase 3 trial of tofersen initiated in clinically presymptomatic SOD1 variant carriers: The ATLAS study. Neurotherapeutics.

[135] Corey D.R. (2017). Nusinersen, an antisense oligonucleotide drug for spinal muscular atrophy. Nat. Neurosci..

[136] Fogacci F., Ferri N., Toth P.P., Ruscica M., Corsini A., Cicero A.F. (2019). Efficacy and safety of mipomersen: A systematic review and meta-analysis of randomized clinical trials. Drugs.

[137] Baldini F., Calderoni M., Vergani L., Modesto P., Florio T., Pagano A. (2021). An overview of long non-coding (lnc) RNAs in neuroblastoma. Int. J. Mol. Sci..

[138] Yang X., Xie Z., Lei X., Gan R. (2020). Long non-coding RNA GAS5 in human cancer. Oncol. Lett..

[139] Yu J., Ou Z., Lei Y., Chen L., Su Q., Zhang K. (2020). LncRNA MYCNOS facilitates proliferation and invasion in hepatocellular carcinoma by regulating miR-340. Hum. Cell.

[140] Lennox K.A., Behlke M.A. (2016). Cellular localization of long non-coding RNAs affects silencing by RNAi more than by antisense oligonucleotides. Nucleic Acids Res..

[141] Rossi J.J., Rossi D.J. (2021). siRNA drugs: Here to stay. Mol. Ther..

